# Nurses' experiences, expectations, and preferences for mind-body practices to reduce stress

**DOI:** 10.1186/1472-6882-11-26

**Published:** 2011-04-11

**Authors:** Kathi Kemper, Sally Bulla, Deborah Krueger, Mary Jane Ott, Jane A McCool, Paula Gardiner

**Affiliations:** 1 Center for Integrative Medicine, Wake Forest University Baptist Medical Center; Winston-Salem, NC, USA; 2 Nursing, Dana-Farber Cancer Institute; Boston, MA, USA; 3 Northeastern University, School of Nursing, Bouve College of Health Sciences, Boston, MA, USA; 4 Family Medicine, Boston University School of Medicine; Boston, MA, USA

## Abstract

**Background:**

Most research on the impact of mind-body training does not ask about participants' baseline experience, expectations, or preferences for training. To better plan participant-centered mind-body intervention trials for nurses to reduce occupational stress, such descriptive information would be valuable.

**Methods:**

We conducted an anonymous email survey between April and June, 2010 of North American nurses interested in mind-body training to reduce stress. The e-survey included: demographic characteristics, health conditions and stress levels; experiences with mind-body practices; expected health benefits; training preferences; and willingness to participate in future randomized controlled trials.

**Results:**

Of the 342 respondents, 96% were women and 92% were Caucasian. Most (73%) reported one or more health conditions, notably anxiety (49%); back pain (41%); GI problems such as irritable bowel syndrome (34%); or depression (33%). Their median occupational stress level was 4 (0 = none; 5 = extreme stress). Nearly all (99%) reported already using one or more mind-body practices to reduce stress: intercessory prayer (86%), breath-focused meditation (49%), healing or therapeutic touch (39%), yoga/tai chi/qi gong (34%), or mindfulness-based meditation (18%). The greatest expected benefits were for greater spiritual well-being (56%); serenity, calm, or inner peace (54%); better mood (51%); more compassion (50%); or better sleep (42%). Most (65%) wanted additional training; convenience (74% essential or very important), was more important than the program's reputation (49%) or scientific evidence about effectiveness (32%) in program selection. Most (65%) were willing to participate in a randomized trial of mind-body training; among these, most were willing to collect salivary cortisol (60%), or serum biomarkers (53%) to assess the impact of training.

**Conclusions:**

Most nurses interested in mind-body training already engage in such practices. They have greater expectations about spiritual and emotional than physical benefits, but are willing to participate in studies and to collect biomarker data. Recruitment may depend more on convenience than a program's scientific basis or reputation. Knowledge of participants' baseline experiences, expectations, and preferences helps inform future training and research on mind-body approaches to reduce stress.

## Background

Stress and burnout are common among nurses, the largest group of health professionals [1-7]. Maintaining a calm, compassionate attitude is a core nursing skill [8-12]. Occupational stress among nurses is important because it can adversely affect attitudes, staff morale, communication, cognition, and quality of care[2,13-15]. Training in mind-body practices, such as meditation, can reduce stress and burnout and improve health outcomes [14,16-25]. Training nurses in mind-body skills could also indirectly improve the quality of care by improving staff health and teamwork, and decreasing unanticipated absences and turnover [19,26-29]. However, little is known about the most effective mind-body practices or training for health professionals in general or nurses in particular, suggesting the need for comparative effectiveness research. Such research should be grounded upon a clear understanding of nurses' baseline experiences, expectations, and preferences for mind-body practices.

According to the US National Institutes of Health (NIH) National Center for Complementary and Alternative Medicine (NCCAM), mind-body practices "focus on the interactions among the brain, mind, body, and behavior, with the intent to use the mind to affect physical functioning and promote health" and include several different practices [30]. For example, intercessory prayers for others' health, which could be considered a mind-body practice, is the most commonly used complementary health therapy in the US[31,32]. Sitting meditation practices such as deep breathing, mindfulness-based stress reduction (MBSR), the Relaxation Response, and Transcendental Meditation™ are also common mind-body practices [33]. Nursing practices such as therapeutic touch and healing touch include a centering component similar to meditation, and explicitly extend compassion and good will, similar to prayer.

Although there has been enormous growth in the number of studies evaluating the health benefits of meditation, the paucity of direct comparisons between training in the different kinds of practices creates a challenge for those planning mind-body training programs to reduce nurses' stress and improve health care quality and outcomes[16,34-39]. Before large comparative effectiveness studies are undertaken, a greater understanding of existing practices and preferences for future training is desirable.

Because mind-body practices are commonly used by the general public, it is likely that some nurses also use them, but few studies have assessed the *prevalence *of mind-body practices and training. Whether or not professionals personally practice mind-body skills, they may have *expectations *about their health benefits which may influence their enrollment in or response to mind-body training programs. However, little is known about nurses' expectations about the health effects of mind-body training. Also, nurses may have *preferences *about the type or format of training which could affect recruitment and retention in training programs, but these factors have not been systematically assessed. Before implementing expensive training programs or undertaking costly studies to compare different kinds of mind-body practices, it would be useful to better understand nurses' experiences with mind-body practices, their expectations about benefits, their preferences for training, and their willingness to participate in research.

The purpose of this study was to prepare for subsequent studies comparing different mind-body approaches to reducing occupational stress among nurses. Because most studies of mind-body training involve voluntary courses that recruit subjects who are interested in stress reduction, a voluntary survey of nurses interested in reducing stress seemed an appropriate first step. The primary study questions were: Among nurses who are interested in stress reduction: 1. What experience do they already have with mind-body practices to reduce stress? 2. In addition to reducing stress, what other health benefits do they expect mind-body approaches to have for them? 3. What factors affect their preferred training?, and 4. Would they be willing to participate in studies of training, be randomized, and provide biomarker data for such studies?

## Methods

To answer these questions, an anonymous, cross-sectional on-line survey was conducted in spring, 2010. A broad response from nurses in a variety of settings was sought with the goal of receiving at least 300 completed surveys from a variety of settings. Nurses were eligible if they practiced in ambulatory or inpatient settings, community or academic settings, and whether they were in training or in practice. Internet access was necessary for participation because recruitment was conducted by email.

Recruitment was conducted solely through email. Approximately 75 email invitations were sent between April and June, 2010 to colleagues, leaders in nursing organizations, and to Listserv groups that included nurses. These included the Directors of Nursing at Wake Forest University Baptist Medical Center; the Directors of the Nursing Magnet program at the 17 North Carolina Magnet Hospitals recognized by the American Nurses' Credentialing Center; a nursing leader at the Ralph H. Johnson Veterans Administration (VA) Medical Center in Charleston, South Carolina; the Director of the Rhode Island State Nurses Association; the Dean of the School of Nursing at the University of New Brunswick; the Boston area coordinator of Therapeutic Touch International Association; and a nursing leader at Kent County Memorial Hospital in Rhode Island. They also included Listservs for Pediatric Integrative Medicine; the North Carolina Mountain Area Health Education Center's Nursing Consortium; and the Association of Wound Specialists.

The emails described the purpose of the survey and provided a link to the Survey Monkey site (SurveyMonkey can be found at http://www.surveymonkey.com. A PDF file of the survey questions is available on request from the authors), the Institutional Review Board (IRB) approval number, contact information for the investigators, and a request to forward the email to other nurses interested in mind-body practices. Due to the nature of the email survey distribution and subsequent email forwarding, it was not possible to determine a denominator for the number of nurses that eventually received an invitation to participate.

The survey was developed, reviewed, and revised by a multidisciplinary group including a meditation teacher, a psychologist with extensive experience with mind-body practices, researchers, nurses, nurse educators, and nursing administrators. It was pilot tested with two experienced nurses in two states before being distributed. In the pilot phase (which did not lead to any substantial revisions), the entire survey required less than 20 minutes to complete. It consisted of 5 e-pages with multiple choice questions: 1) previous experiences, training and practice with meditation, prayer, and other mind-body practices included in the NIH NCCAM category of mind-body practices as well as nursing biofield practices of therapeutic and healing touch (Although healing touch and therapeutic touch are generally considered biofield therapies, they were included in this survey at the suggestion of nurses who view them as ways of centering and extending compassion that reduce stress in providers as well as patients.); 2) expectations about expected benefits of meditation practice for physical, emotional, mental, spiritual, and social health; 3) respondents' overall health status, occupational stress, and presence of one or more common health conditions; 4) demographic characteristics, practice location, and current involvement in research; and 5) preferences about type and format of meditation training, willingness to be randomized in comparison studies, and willingness to collect biomarker data. Answers were multiple choice and provided space for respondents to make comments.

Because the purpose of this study was to describe nurses' experiences and attitudes, data analysis relied on simple descriptive statistics. The anonymous data were downloaded from Survey Monkey into an Excel spreadsheet and exported to SAS version 9.1 for analysis.

This study was approved by the Wake Forest University Health Sciences Institutional Review Board (IRB).

## Results

### Subject characteristics

Between April 15, 2010 when the survey was approved by the IRB and June 30, 2010 when enrollment was closed, 342 nurses responded to the survey, of which 96% were women. Most (92%) were Caucasian, 4% were African American, 2% were mixed/other, 1% were Latino, and 1% were Asian. Most (63%) were more than 45 years old, and 80% had been in practice for 10 or more years. Most (62%) were registered nurses (RNs), nurses with masters or doctoral degrees (33%), or nurses' aides, licensed practical nurses (LPNs) or licensed vocational nurses (LVNs) (5%). Respondents lived in all major regions of the US designated by the National Health Interview Survey (NHIS): 58% from the southern US, 17% from the northeast, 11% from the west, 4% from the midwest; and 11% were Canadians.

The respondents practiced in a variety of settings: 36% practiced in academic health centers in inpatient settings, 26% in academic ambulatory settings, 19% in community outpatient or ambulatory settings, 11% in community inpatient settings (including long-term care, nursing homes, and hospice), and 9% in other settings.

Most (91%) nurses reported having excellent (20%), very good (41%), or good (30%) overall health. Of the 73% who reported one or more health conditions, the most common were anxiety (49%), back pain (41%), GI problems such as irritable bowel syndrome and reflux (34%), and depression (33%) (Table 1).

**Table 1:** Health conditions in the past 12 months (more than one answer allowed)

Health Conditions in Past Year	Percentage of nurses who reported this condition
Anxiety	49
Back pain	41
GI Problems such as IBS or reflux severe enough to interfere with work	34
Depression	33
Arthritis	24
High blood pressure or Heart Disease	21
Headaches severe enough to interfere with work	19
Asthma	9
Diabetes	6
Chronic pain or fibromyalgia	3
Cancer or cancer survivor	2

**Figure 1:**
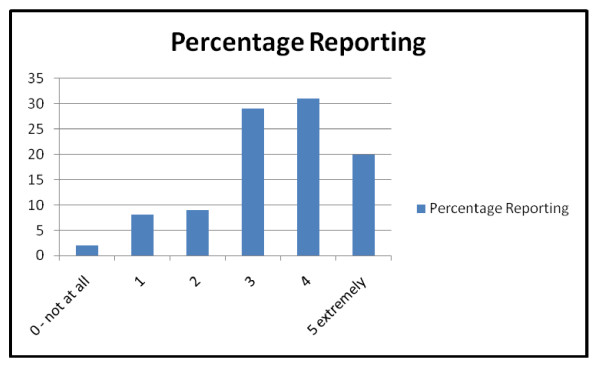
**Stress levels in past 12 months in primary work location**.

On a scale from 0 (not at all stressed) to 5 (extremely stressful), nurses' reported a median stress level 4 in their primary work environment over the past 30 days (Figure 1).

### Experiences with Mind-Body Practices to Reduce Stress

Nearly all (99%) nurses reported one or more mind-body practices in the previous 12 months. The most common mind-body practices were prayer-based (Table 2). Specifically, over 85% of nurses reported having prayed for another person's health. In comparison, concentration-type meditation such as Relaxation Response or Transcendental Meditation practices were reported by 23%, and mindfulness-based meditation was reported by 18%. Other common mind-body practices included providing healing touch or therapeutic touch (39%), meditative movement such as yoga, tai chi or qigong (34%), and guided imagery or hypnosis (25%).

**Table 2:** Nurses' experiences with mind-body practices in past 12 months

Prayer practices	Percentage Practicing
Intercessory (for someone else's health or well-being)	86
Prayers of forgiveness, gratitude, or thanksgiving	82
Prayers for peace, harmony, understanding between people	65
Praise or devotion	52
Centering or grounding prayer	40
Prayerful singing	34
Reading prayers, daily devotional or sacred texts	32
Rosary	8
*NO PRAYER practices in past 12 months*	*6*
**Meditation Practices**	
Breath-focused	49
Visualization-based (object, mandala, condition)	26
Compassion or lovingkindness	25
Concentration-type (including Relaxation Response and TM)	23
Affirmation-based	22
Contemplative	18
Mindfulness-based (includes MBSR, Vipassana)	18
Sound-based (chanting or mantra-based)	15
Zen	3
*NO MEDITATION practices in past 12 months*	*35*
**Other Mind-Body Practices**	
Healing Touch or Therapeutic Touch	39
Yoga, Tai Chi, QiGong, or other mindful movement	34
Guided Imagery or Hypnosis	25
Reiki, Polarity therapy, or other mindful energy healing	21
Biofeedback to promote relaxation or well-being	6
Autogenic Training	3
Other (massage, acupuncture, crystals)	2
*NO OTHER Mind-Body Practices*	*29*
**No Mind-Body Practices (Prayer, Meditation, or Other Mind-Body Practices) in Past 12 months**	*1*

**Figure 2:**
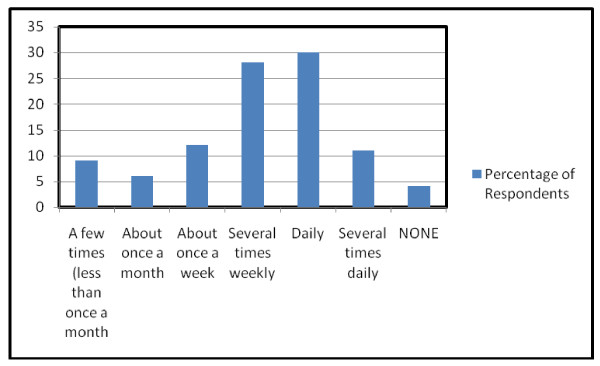
**Frequency of mind-body practices**.

Nurses typically engaged in a mind-body practice daily or several times weekly for less than 20 minutes per session (Figure 2). Most (62%) typically practiced alone, while the rest practiced sometimes or only in groups. Nurses reported receiving several types of training, such as group training/class (42%), reading a book or web site (37%), listening to a CD/MP3 or watching a DVD or YouTube video (24%), individual training with a teacher (17%), or on-line training (4%); some (8%) nurses reported that they were already teachers of one or more mind-body practices.

### Expected benefits from mind-body training

Nurses expected a variety of health benefits from additional training in mind-body practices (Table 3). At least 20% of nurses expected a great (vs. moderate, little, or no) expected benefit for every item listed on the survey. The items most commonly endorsed as having great expected benefit were more often emotional or spiritual than physical, mental, or social. For example, more than 50% of respondents expected great benefits for more serenity, less anxiety, or greater spiritual well-being, inner peace, or connection with God or a higher power. In contrast, fewer than 50% of nurses expected great benefits for pain, sleep, or being more effective in their professional or personal relationships.

**Table 3:** Expected physical, emotional, mental, spiritual, and social benefits of meditation training for nurses (more than one response allowed)

Physical benefits	% of respondents expecting GREAT benefit
More resilience in the face of physical challenges	42
Sleep better	42
Overall better physical health	41
Energy or vitality better (less fatigue)	37
Strong immunity	36
Pain less/comfort greater	33
Blood pressure lower	29
Weight better	21
**Emotional benefits**	
More serenity/calmness	54
Less anxiety or worry	53
Better mood	51
More happiness or cheerfulness	46
Less burned out, discouraged, or cynical	46
More emotional resilience	46
More confidence or courage	44
More accepting	43
**Mental benefits**	
More mindful - being more present in each moment	48
Overall better mental health	44
Better intuition	40
Greater clarity	39
Better focus or concentration	39
More creative	37
Less judgmental	37
Greater discernment	35
Less distractible	31
Better memory	26
Faster thinking	26
**Spiritual benefits**	
Greater spiritual well-being	56
More inner peace	54
Greater connection with God or Higher Power	53
More compassionate or loving	50
More forgiving	48
Greater coherence (sense that life is comprehensible and meaningful)	46
More wisdom	44
Greater appreciation for nature	42
**Social benefits**	
Greater kindness	44
Better listener	41
More effective in my professional work	40
More empathetic	39
Better relationship with my patients	37
Better family relationships	36
More generous	35
Better relationships with my team	32
Stronger friendships	31
Better communication with others	31
Stronger social support	28
More social connections	27
Better relationship with my supervisor	27
Better able to ask for and receive help from others	25

NOTE: The question was " how much benefit do you EXPECT that training in meditation would have for you personally?" Responses included None, A little, Moderate, or Great benefit. For simplicity, this table lists the percentage of respondents for each item who reported Great benefit.

### Preferences for Training and Willingness to Participate in Research

Over 90% of nurses reported interest in receiving additional mind-body training. When given choices between in-person or electronic training methods, the most commonly chosen was in-person (45%), followed by DVD/CD/MP3 (37%), with webinar (18%) as the least preferred training method. However, convenience was cited by 74% as being essential or very important in choosing a future training program. The time required to complete training (58%), time required for daily practice (60%), and being able to train at one's own pace (58%) were also essential or very important in choosing training. Getting to know the instructor, the teacher's or program's reputation, and the scientific evidence for a program's effectiveness were all less important (Table 4).

**Table 4:** Preferences for training

Factors Affecting Training Preferences	Percentage Reporting Very important or Essential
Convenience	74
Time required for daily practice	61
Time commitment to complete training	59
Doing it at my own pace	59
Reputation of sponsoring institution	49
Reputation of teacher	47
Reinforcing or strengthening an existing skill or practice	42
Privacy	42
Consistent with my religious beliefs	35
Scientific studies supporting a particular practice	32
Introductory training	30
Getting to know the teacher better	19
Group training in person	16
Intensive training	13
Novelty (new type of practice for me)	13
Being part of a group	12

NOTE: Responses included -- not at all important; somewhat important; moderately important; very important; or essential. For simplicity, this table lists the percentage of respondents who reported very important/essential (combined).

Although most (65%) of those willing to enroll in a research-related training program were also willing to be randomized, 35% had such strong preferences for type or format of training that they were unwilling participate in a study requiring randomization.

Of potential control interventions for a future study of sitting meditation, those of most interest were yoga or tai chi (52%), massage (46%), and acupuncture (36%). Fewer nurses were interested in control groups featuring advice or education about diet/nutrition (26%), exercise/fitness (27%), or natural health products (24%).

Although nurses primarily expected strong benefits for emotional and spiritual well-being, 84% of those willing to participate in a study were willing to have at least one biomarker collected before and after training to assess the impact of training. Most were willing to have weight measured (62%); collect their own saliva for cortisol measurement up to 4 times daily (60%); have their blood pressure (BP) measured (54%); have an electrocardiogram (ECG) reading to determine heart rate variability (56%); and/or blood drawn for biomarkers (53%).

## Discussion

This is the first study to provide a detailed description of nurses' experience with, expectations of, and preferences for practices and training in mind-body approaches to reducing stress. These factors affect recruitment to, retention in, and impact of mind-body training programs [40-42]. They have important implications for those planning or evaluating mind-body training programs to reduce stress among health professionals.

This study focused on nurses because they are the largest group of health professionals; they often experience stress; and stress can adversely affect their personal health as well as the quality and cost of care they provide. The survey included a large number of nurses practicing in a variety of settings across North America. The results are consistent with earlier studies showing high rates of occupational stress and personal health conditions frequently related to stress such as anxiety, back pain, functional bowel disorders, and depression [2,5,43-48]. Future studies may use similar methodology to assess the experiences, expectations, and preferences of other health professionals interested in using mind-body practices to reduce occupational stress.

These results are also consistent with other surveys in which nurses had positive attitudes about mind-body therapies, were already using one or more of them, and wanted additional training [49-53]. For example, many critical care nurses personally used relaxation therapy (87%), therapeutic touch (83%), prayer (84%), and meditation (63%), and were interested in additional training [50]. Similarly, the complementary therapies most often used by the clinical nurse specialists in Minnesota included spirituality/prayer (71%), relaxed breathing (57%), and meditation (34%) [48]. The data from this study are unique in surveying North American nurses in a variety of settings who are interested in additional mind-body training, and eliciting information about a very broad range of potential practices. Our survey shows that nearly all nurses interested in mind-body training to reduce stress already practice one or more mind-body strategies. This suggests that future studies evaluating the impact of mind-body training should conduct stratified analyses to control for baseline experience and expectations.

The choice of mind-body practices to include in the survey was informed by discussion with nurses and included healing touch, therapeutic touch, and prayer as well as meditation, hypnosis, and yoga. A number of training programs teach nurses to provide therapeutic touch or healing touch, which NCCAM currently categorizes as biofield therapies. Central to both therapeutic and healing touch are the practices of centering and intentionally extending calm, caring compassion which appear to reduce stress and improve overall well being among the nurses who learn them [29,54]. Several different types of prayer were included because it is so commonly practiced as a way of coping [31]. Furthermore, the US Joint Commission mandates the assessment of patients' spiritual needs, so explicit attention to this arena is an integral aspect of nursing practice. The number of nurses using prayer as a stress management strategy exceeded our expectation; the relative proportion of professionals using different strategies may vary geographically, culturally, and by age, race, and/or profession.

The nurses in this study reported numerous expectations about the expected benefits of mind-body training on physical, emotional, mental, spiritual, and social well-being as well as stress. It was not an intervention study and did not assess the actual impact of any mind-body practice. Nurses primarily expected greater benefits in terms of spiritual well-being (56%), inner peace (54%), or serenity (54%) compared with physical outcomes such as better sleep (42%), immunity (36%), or blood pressure (29%). This information builds on results from earlier surveys in which nurses expected that complementary therapies would be helpful with a variety of physical and mental concerns including anxiety, pain, and insomnia [50,55,56]. Matching recruitment materials and outcome measures with nurses' expectations about benefits may improve recruitment and retention in future training programs. Biomarkers alone may be insufficient to capture the range of expected benefits of mind-body training.

Over 90% of nurses in this study were interested in additional training despite a high rate of existing practice. The information about factors affecting interest in participation (e.g., convenience and time required for training and practice as more important than established effectiveness or reputation) could help when planning and recruiting for training programs. Furthermore, information about preferences for in-person vs. electronic training methods can assist in planning future interventions.

Although most nurses were willing to participate in research on mind-body training, 35% were unwilling to be randomized, suggesting that a combination of RCTs and preference or cohort trials may be useful. The most frequently preferred comparison interventions (compared with sitting meditation practices) were yoga, Tai Chi or QiGong. Comparing sitting with movement-based meditative practices would be useful because movement-based practices may have additional benefits associated with exercise [57-62]. Finally, this study suggests that even though nurses have the strongest expectations about spiritual and emotional benefits of meditation, over 80% are willing to collect one or more kinds of biomarker data. However, they are less willing to have blood drawn than to be weighed or collect salivary cortisol.

As a survey of self-selected nurses, this study has several limitations. Just as only a subset of eligible subjects enroll in evaluations of mind-body training, only a subset of nurses respond to a survey on mind-body practices, so interest and practices in this survey may overestimate experience and expectations in the general nursing profession. On the other hand, the survey specifically sought responses from nurses interested in mind-body training to reduce stress, so it is likely to build a better platform for research recruiting voluntary recruits for studies of mind-body training than studies that ask nurses who may not be interested in stress reduction training. Because nurses were recruited by email, response rate cannot be calculated. The respondents included few ethnic or racial minorities; all had access to email and were able to complete an on-line survey in English, limiting generalizability. One the other hand, the survey was completed by a large number of nurses from diverse geographic locations and practice locations, increasing the likelihood that these results would be meaningful in different settings. This study did not directly assess the impact of mind-body practices, but it prepares the way for comparative effectiveness research on mind-body interventions. As a descriptive study, analyses to determine what factors predict which nurses would be interested in what types of mind-body training were not conducted. This would be a worthwhile question for future research. Finally, additional research is needed to understand nurses' perspectives on mind-body training when provided in the context of mandatory or required courses compared with elective formats.

## Conclusions

This study confirms earlier research suggesting that many nurses experience high levels of work-related stress, and many already have personal experience with mind-body practices. The most commonly used practices to manage stress include prayer, breath-focused meditation, and healing touch/therapeutic touch. Nurses expect these practices to have spiritual, emotional, and mental as well as physical benefits; want training that is convenient; and are willing to participate in and collect biomarker data for comparative effectiveness research,. These results inform future projects in mind-body training and research.

## Competing interests

The authors declare that they have no competing interests.

## Authors' contributions

KK conceived of and drafted the survey, participated in data analysis, drafted and revised the manuscript.

DK, SB, MJO, and JM reviewed the survey instrument, disseminated the survey to nurses, reviewed and participated in revising the manuscript.

PG analyzed the data, reviewed and participated in revising the manuscript.

All authors read and approved the final manuscript.

## Pre-publication history

The pre-publication history for this paper can be accessed here:

http://www.biomedcentral.com/1472-6882/11/26/prepub
